# Simulated Microgravity and Recovery-Induced Remodeling of the Left and Right Ventricle

**DOI:** 10.3389/fphys.2016.00274

**Published:** 2016-06-29

**Authors:** Guohui Zhong, Yuheng Li, Hongxing Li, Weijia Sun, Dengchao Cao, Jianwei Li, Dingsheng Zhao, Jinping Song, Xiaoyan Jin, Hailin Song, Xinxin Yuan, Xiaorui Wu, Qi Li, Qing Xu, Guanghan Kan, Hongqing Cao, Shukuan Ling, Yingxian Li

**Affiliations:** ^1^State Key Laboratory of Space Medicine Fundamentals and Application, China Astronaut Research and Training CenterBeijing, China; ^2^Key Laboratory of Molecular and Cellular Biology of Ministry of Education, College of Life Science, Hebei Normal UniversityShijiazhuang, China; ^3^State Key Laboratory of Agrobiotechnology, College of Life Sciences, China Agricultural UniversityBeijing, China; ^4^Medical Experiment and Test Center, Capital Medical UniversityBeijing, China

**Keywords:** simulated microgravity, cardiac remodeling, declining function, recovery, remodeling pathways

## Abstract

Physiological adaptations to microgravity involve alterations in cardiovascular systems. These adaptations result in cardiac remodeling and orthostatic hypotension. However, the response of the left ventricle (LV) and right ventricle (RV) following hindlimb unloading (HU) and hindlimb reloading (HR) is not clear and the underlying mechanism remains to be understood. In this study, three groups of mice were subjected to HU by tail suspension for 28 days. Following this, two groups were allowed to recover for 7 or 14 days. The control group was treated equally, with the exception of tail suspension. Echocardiography was performed to detect the structure and function changes of heart. Compared with the control, the HU group of mice showed reduced LV-EF (ejection fraction), and LV-FS (fractional shortening). However, mice that were allowed to recover for 7 days after HU (HR-7d) showed increased LVIDs (systolic LV internal diameter) and LV Vols (systolic LV volume). Mice that recovered for 14 days (HR-14d) returned to the normal state. In comparison, RV-EF and RV-FS didn't recover to the normal conditions till being reloaded for 14 days. Compared with the control, RVIDd (diastolic RV internal diameter), and RV Vold (diastolic RV volume) were reduced in HU group and recovered to the normal conditions in HR-7d and HR-14d groups, in which groups RVIDs (systolic RV internal diameter) and RV Vols (systolic RV volume) were increased. Histological analysis and cardiac remodeling gene expression results indicated that HU induces left and right ventricular remodeling. Western blot demonstrated that the phosphorylation of HDAC4 and ERK1/2 and the ratio of LC3-II / LC3-I, were increased following HU and recovered following HR in both LV and RV, and the phosphorylation of AMPK was inhibited in both LV and RV following HU, but only restored in LV following HR for 14 days. These results indicate that simulated microgravity leads to cardiac remodeling, and the remodeling changes can be reversed. Furthermore, in the early stages of recovery, cardiac remodeling may be intensified. Finally, compared with the LV, the RV is not as easily reversed. Cardiac remodeling pathways, such as, HDAC4, ERK1/2, LC3-II, and AMPK were involved in the process.

## Introduction

There are various changes in the human cardiovascular system, including a cephalic fluid shift (Thornton et al., 1987), changes in cardiac systolic volume (Bungo et al., 1987; Caiani et al., 2006), and over time, a loss of left ventricular mass due to microgravity during space flight (Perhonen et al., 2001; Summers et al., 2005). The adaptation and adjustments that characterize the responses to the metabolic demands of activity and gravitational loading on Earth are changed dramatically under conditions of microgravity. Chronic reduction in metabolic demand and oxygen uptake reduces the demand on cardiac output and tissue perfusion, resulting in cardiac atrophy and a decline in function, and further leads to orthostatic intolerance upon return to full gravity with the potential risk of irreversible structural changes that may become pathological (Marcus et al., 1977; Zile et al., 1993; Perhonen et al., 2001). Because of this, it is essential to determine the severity of cardiac changes upon return to the ground. Many studies have demonstrated that cardiac remodeling induced by microgravity and/or simulated microgravity is associated with a decline in cardiac function. However, the changes in heart structure and function during reloading following simulated microgravity are not well-understood.

An abundance of data has provided insight into the changes that occur in the left ventricle (LV; Summers et al., 2005; Westby et al., 2016). There are no data, however, on remodeling in the right ventricle (RV) under weightlessness due to reduced gravitational loading. The changes in the LV and RV that occur in astronauts during space flight and their subsequent return to the ground are poorly understood. On the cellular level, the remodeling responses of the LV and RV to pressure overload are largely similar. There are several major signaling molecules involved in cardiac remodeling induced by external or intrinsic stimuli, including HDAC4, AMP-activated protein kinase (AMPK), ERK1/2, and LC3-II. However, there is a divergence in the molecular mechanisms of the RV compared with the LV under stress conditions (Reddy and Bernstein, 2015). The difference in the responses of the LV and RV to simulated microgravity as well as the signaling molecules involved in this process need to be explored further.

From the perspective of the cardiovascular system, rodent hindlimb unloading (HU) is a suitable model. There is extensive literature investigating the cardiovascular adaptation to simulated microgravity, predominantly using the HU rat or mouse model (Hasser and Moffitt, 2001). The mouse demonstrates a wide range of cardiovascular responses to HU-simulated microgravity, including alterations in heart function, heart rate, exercise capacity, peripheral arterial vasodilatory responsiveness, and the baroreflex response (Powers and Bernstein, 2004). Many of these responses are similar to those seen in humans. Following 28 days of HU-simulated microgravity, mice manifest many of the cardiovascular alterations that have been previously demonstrated in humans during space flight (Buckey et al., 1996; Fritsch-Yelle et al., 1996; Powers and Bernstein, 2004).

Here, we suppose that HU can lead to distinct remodeling of the LV and RV in mice, and that reloading after HU has a further effect on left and right ventricular remodeling. In this study, we detected the remodeling signals and structural changes of the LV and RV following HU and hindlimb reloading (HR). We determined that pathological remodeling signals are overactive in both the LV and RV following HU and/or HR, and are restored after 14 days of reloading. The physiological remodeling signal AMPK is downregulated in both the LV and RV, which leads to the functional decline of both ventricles. Finally, we found that recovery is more difficult in the RV than the LV. This study provides insight into the molecular mechanisms of cardiac remodeling and the decline of systolic function of both the LV and RV during simulated microgravity and recovery.

## Materials and methods

### Animals

All mice used in the experiments were bred and maintained at the SPF Animal Research Building of China Astronaut Research and Training Center (12-h light, 12-h dark cycles, temperature controlled for 23°C and free access to food and water). The mice used on this study were 3 month old males and in a C57BL/6N background. The experimental procedures were approved by the Animal Care and Use Committee of China Astronaut Research and Training Center, and all animal studies were performed according to approved guidelines for the use and care of live animals.

### Hindlimb-unloading model

The hindlimb-unloading procedure was achieved by tail suspension, as described by Morey-Holton and Globus (2002). Briefly, the 3-month-old mice were individually caged and suspended by the tail using a strip of adhesive surgical tape attached to a chain hanging from a pulley. The mice were suspended at a 30° angle to the floor with only the forelimbs touching the floor, which allowed the mice to move and access to food and water freely. The mice were subjected to hindlimb unloading through tail suspension for 28 days, which we will identify as the “unloaded” state, for a total of 28 days, after which they were returned to the normal four-extremity weight bearing “reloaded” position (hindlimb reloading, HR). Similar numbers of control mice of the same strain background were instrumented and monitored in similar fashion under identical cage conditions but without tail suspension.

### Histological analysis

Sections were generated from paraffin embedded hearts, and were stained with H&E for gross morphology, Masson's trichrome for detection of fibrosis, as described before Ling et al. (2012).

### RNA extraction and real-time polymerase chain reaction

Total RNA was extracted from heart tissues by using RNAiso Plus reagent (Takara) according to the manufacturer's protocol. The RNA (500 ng/sample) was reverse transcribed into cDNA and qPCR was performed using a SYBR Green PCR kit (Takara) in a Light Cycler (Eppendorf, Germany). PCR for each sample was carried out in duplicate for all cDNAs. The mRNA level of each gene was normalized to that of *Gapdh*, which served as an internal control. Primers (synthesized by Sunbiotech Co, China) for *Col1a1* (Product size, 119 bp, Melting temperature, 61°C), *Col3a1* (Product size, 98 bp, Melting temperature, 60°C), BNP (Product size, 185 bp, Melting temperature, 56°C), and *Gapdh* (Product size, 122 bp, Melting temperature, 59°C), were as follows:

*Col1a1* sense primer: 5′-CTG ACTGGAAGAGCGGAGAGT-3′,
*Col1a1* anti-sense primer: 5′-AGA CGGCTGAGTAGGGAACAC-3′;
*Col3a1* sense primer: 5′-ACG TAAGCACTGGTGGACAG-3′,
*Col3a1* anti-sense primer: 5′- CAGGAGGGCCATAGCTGAAC-3′;
BNP sense primer: 5′-TGT TTCTGCTTTTCCTTTATCTG-3′,
BNP anti-sense primer: 5′-TCT TTTTGGGTGTTCTTTTGTGA-3′;
*Gapdh* sense primer: 5′-ACT CCACTCACGGCAAATTCA-3′,
*Gapdh* anti-sense primer: 5′-GGC CTCACCCCATTTGATG-3′.

### Transthoracic echocardiography

Animals were lightly anesthetized with 2,2,2-tribromoethanol (0.2 ml/10 g body weight of a 1.2% solution) and set in a supine position. Two dimensional (2D) guided M-mode echocardiography was performed using a high resolution imaging system (Vevo 770, Visual-Sonics Inc., Toronto, ON, Canada). Two-dimensional images are recorded in parasternal long- and short-axis projections with guided M-mode recordings at the midventricular level in both views. Left ventricular (LV) cavity size and wall thickness are measured in at least three beats from each projection. Averaged LV wall thickness [interventricular septum (IVS) and posterior wall (PW) thickness] and internal dimensions at diastole and systole (LVIDd and LVIDs, respectively) are measured. LV fractional shortening ((LVIDd – LVIDs)/LVIDd), relative wall thickness [(IVS thickness + PW thickness)/LVIDd], and LV mass [LV Mass = 1.053 × [(LVIDd + LVPWd + IVSd)3 – LVIDd3]] are calculated from the M-mode measurements. LV ejection fraction (EF) was calculated from the LV cross-sectional area (2-D short-axis view) using the equation LV %EF = (LV Vold – LV Vols)/LV Vold × 100%. For RV, Two-dimensional images are recorded in right parasternal long- and short-axis projections with guided M-mode recordings at the maximum diameter level in both views. Right ventricular (RV) cavity size and wall thickness are measured in at least three beats from each projection. Averaged Right Ventricle Anterior Wall and internal dimensions at diastole and systole (RVIDd and RVIDs, respectively) are measured. Right Ventricle Percent Fractional Shortening (RVIDd – RVIDs)/RVIDd × 100%). Right Ventricle Percent Ejection Fraction (EF) was calculated from the RV cross-sectional area (2-D short-axis view) using the equation RV %EF = (RV Vold – RV Vols)/RV Vold × 100%.

For the RV mass weight calculation, firstly, we measured the RV endocardial borders in diastole (five measures) and systole (five measures) in five consecutive cardiac cycles in each flat-image, generating 10 RV endocardial areas (RVendo). Then, we got the epicardial borders and corresponding RV epicardial areas (RVepi) on the same frames. Ten RV free wall areas were then calculated by subtracting RVendo from RVepi (RVepi-RVendo). Finally, the total RV free wall volume of each plane was calculated by Simpson's method using the mean of the RV free wall area. RV free wall mass was obtained by multiplying this volume by the specific density of the myocardium (1.05 g/cc).

### Western blot analysis

Mouse hearts were crushed by homogenizer (Power Gen125, Fisher Scientific) and then lysed in lysis buffer (50 mM Tris, pH7.5, 250 mM NaCl, 0.1% SDS, 2 mM dithiothreitol, 0.5% NP-40, 1 mM PMSF, and protease inhibitor cocktail) on ice for 15 min. Protein fractions were collected by centrifugation at 12,000 g at 4°C for 15 min and then applied to 8–12% SDS-PAGE gels, electrophoresed at 80 V fof 30 min and 120 V for 90 min. After electrophoresis, protein was transfected to a polyvinylidene fluoride membrane using a Criterion blotter apparatus (Bio Rad). The membrane was then blocked in 5% non-fat dry milk (Becton, Dickinson, and Company) in TBST (10 mM Tris–Cl, 150 mM NaCl, 0.05% Tween-20, pH 7.5) for 1 h. After that, the membrane was incubated with primary antibody overnight at 4°C followed by incubation with a secondary antibody conjugated to horseradish peroxidase (HRP), and visualized using an chemiluminescence kit (Thermo Pierce, No.32 109). Specific antibodies to p-HDAC4 (Cell Signaling Technology, #3443S), HDAC4 (Cell Signaling Technology, #5392S), p-AMPKα (Cell Signaling Technology, #2531S), AMPKα (Cell Signaling Technology, #2532), p-ERK1/2 (Cell Signaling Technology, #4370S), ERK1/2 (Cell Signaling Technology, #4696S), LC3-II/I (MBL, PM036-PN), mTOR (Cell Signaling Technology, #2983), p-mTOR (Cell Signaling Technology, #5536) Atrogin1 (ECM biosciences, # AP2041), MuRF1 (ECM biosciences, # MP3401), Gapdh (Santa Cruz Biotechnology, sc-25778) were used to detect protein levels. Gapdh was used as a loading control.

### Statistical analysis

Data are presented as mean ± SEM per experimental condition. For the statistical differences among groups, considering the presence of unequal variance for the data, we firstly test the equality of variances across groups. If it shows the variances are unequal, we then use Welch's *t*-test for one-way analysis. Otherwise we use Student's *t*-test. Bonferroni adjustment was used for multiple comparisons. *P* < 0.05 is considered statistically significant. *P* < 0.01 is considered very significant. All the statistical tests are analyzed by Prism software (Graphpad prism for windows, version 5.01).

## Results

### Heart weight and body weight changes following HU and recovery

**Figure 1:**
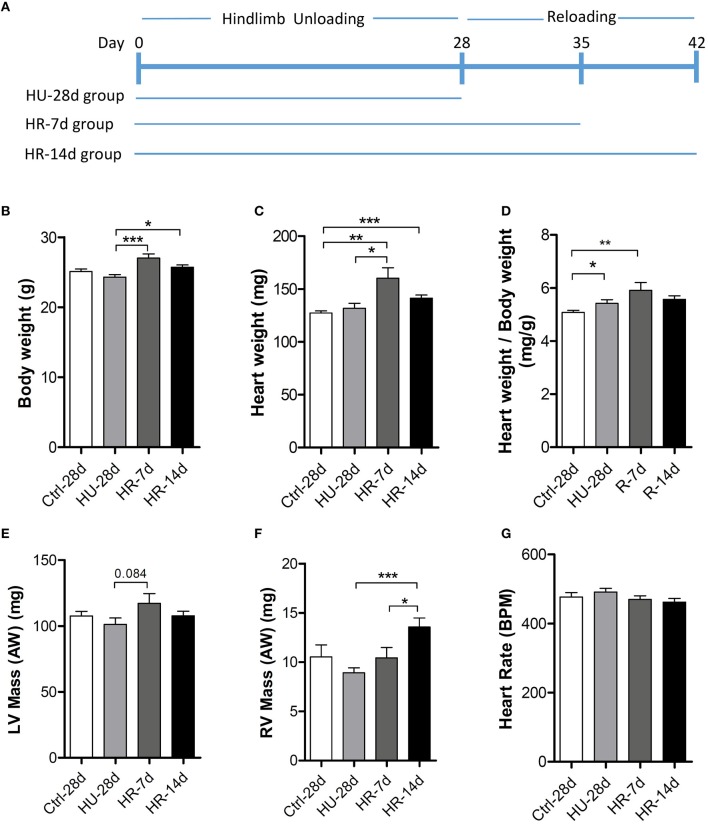
**Heart weight analysis following hindlimb unloading and recovery. (A)**, Timeline summarizing experimental design. Body weight **(B)**, heart weight **(C)**, ratio of heart weight to body weight **(D)**, LV mass **(E)**, RV mass **(F)**, and heart rate **(G)** of mice following hindlimb unloading (HU) and recovery. Ctrl-28d, control mice; HU-28d, mice following 28 days of HU; HR-7d, mice following 7 days of recovery after HU; HU-14d, mice following 14 days of recovery after HU. Values are means ± SEM. ^*^P < 0.05, ^**^P < 0.01, ^***^P < 0.001.

Three groups of mice were subjected to HU by tail suspension for 28 days following which two groups were allowed to recover for 7 or 14 days (HU-28d, *n* = 10; HR-7d, *n* = 10; HR-14d, *n* = 10). The control group (*n* = 10) was treated equally, with the exception of tail suspension (Figure 1A). Body weight and heart weight were recorded before sacrifice (Figures 1B,C), and the mass of the LV and RV were calculated by echocardiography (Figures 1E,F). Compared with the control group, body weight showed an overall decrease, while heart weight increased following HU and HR. Thus, the ratio of heart weight to body weight increased following 28 days of HU and 7 days of HR (Figure 1D). LV mass calculated by echocardiography remained unchanged following HU or HR (Figure 1E), but RV mass exhibited an overall decrease following HU, and recovered following HR (Figure 1F). All echocardiographic measurements were made while mice maintained heart rates of 450 ± 50 beats per minute (Figure 1G).

### Changes in left and right ventricular function following HU and recovery

**Figure 2:**
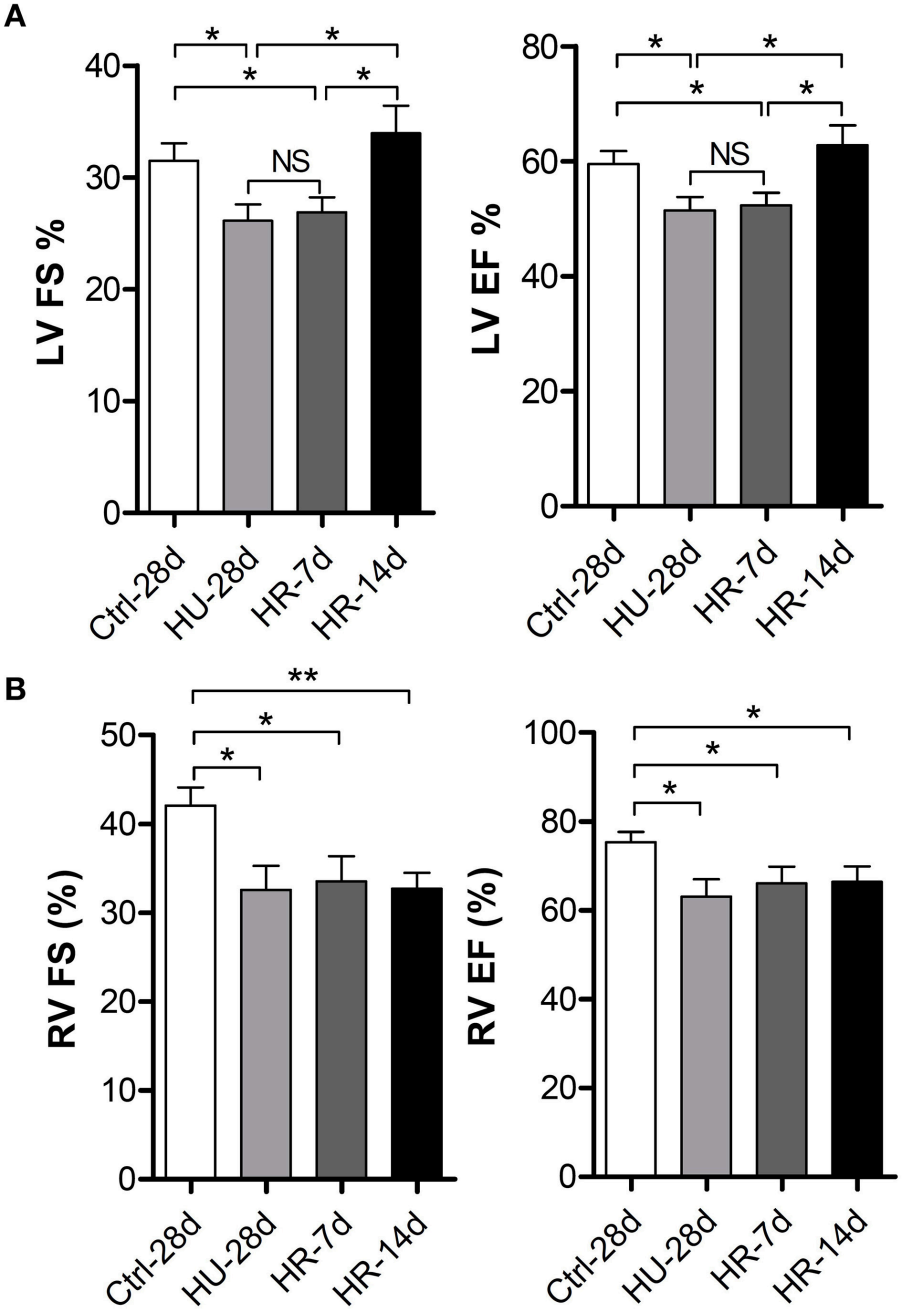
**Changes in left and right ventricular function following hindlimb unloading and recovery. (A)** Echocardiographic assessment of fractional shortening (FS) and ejection factor (EF) of the left ventricle (LV) of mice following HU and recovery. **(B)** Echocardiographic assessment of fractional shortening (FS) and ejection factor (EF) of the RV of mice following HU and recovery. Values are means ± SEM. ^*^P < 0.05, ^**^P < 0.01.

To validate the effects of HU and HR on the LV and RV, transthoracic echocardiography was performed to determine ventricular function following HU and HR. After 28 days of HU, left ventricular fractional shortening (LV-FS), and left ventricular ejection fraction (LV-EF) decreased significantly in HU mice compared with the control, and did not recover during the first few days of HR. Full recovery was only apparent after reloading for 14 days (Figure 2A). However, after 28 days of HU, RV-FS and RV-EF decreased significantly in HU mice compared with the control, and did not recover even when reloaded for 14 days (Figure 2B). The results indicate that simulated microgravity can induce a decline in left and right ventricular function, and that recovery is slower in the RV after reloading.

### Changes in left and right ventricular structure following HU and recovery

**Figure 3:**
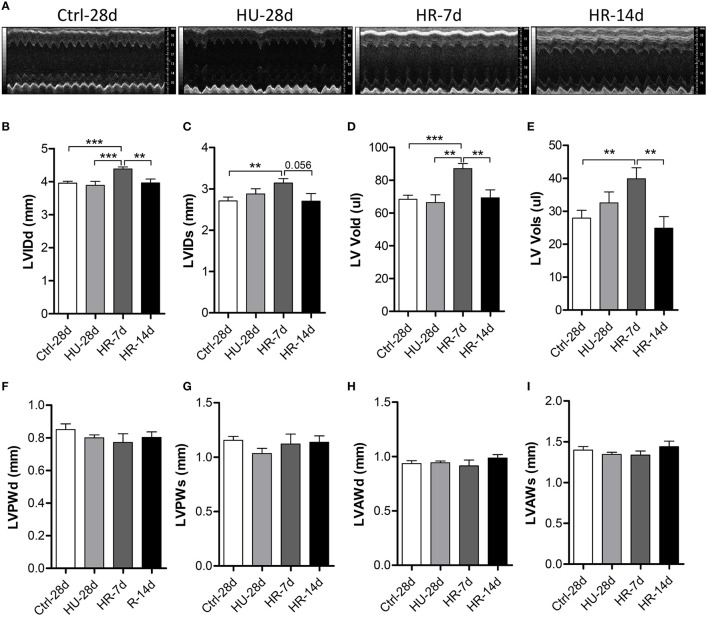
**Transthoracic echocardiography evaluating the left ventricular structure of mice following hindlimb unloading and recovery. (A)** Representative M-mode recordings of echocardiography. **(B–I)** Quantitative analysis of the diastolic and systolic left ventricular internal diameter (LVIDd and LVIDs), LV volume (LV Vold and LV Vols), LV anterior wall thickness (LVAWd and LVAWs), and LV posterior wall thickness (LVPWd and LVPWs) of mice by echocardiography following hindlimb unloading and recovery. Values are means ± SEM. ^**^P < 0.01, ^***^P < 0.001.

To validate the influence of HU and HR in the LV, we performed transthoracic echocardiography to determine the structure of the LV following HU and HR (Figure 3A). Compared with control, the end-systolic LV internal diameter (LVIDs), and the end-diastolic LV internal diameter (LVIDd) did not change in the HU-28d mice, but increased following HR for 7 days, and recovered after 14 days of HR (Figures 3B,C). Furthermore, the change in end-systolic LV volume (LV-Vols) and the end-diastolic LV volume (LV-Vold) was the same as for LVIDs and LVIDd (Figures 3D,E). The LV posterior wall thickness (LVPW) and the LV anterior wall thickness (LVAW) didn't change following HU or HR (Figures 3F–I). Together, these data show that reloading after HU can induce enlargement of the left ventricular internal diameter and volume.

**Figure 4:**
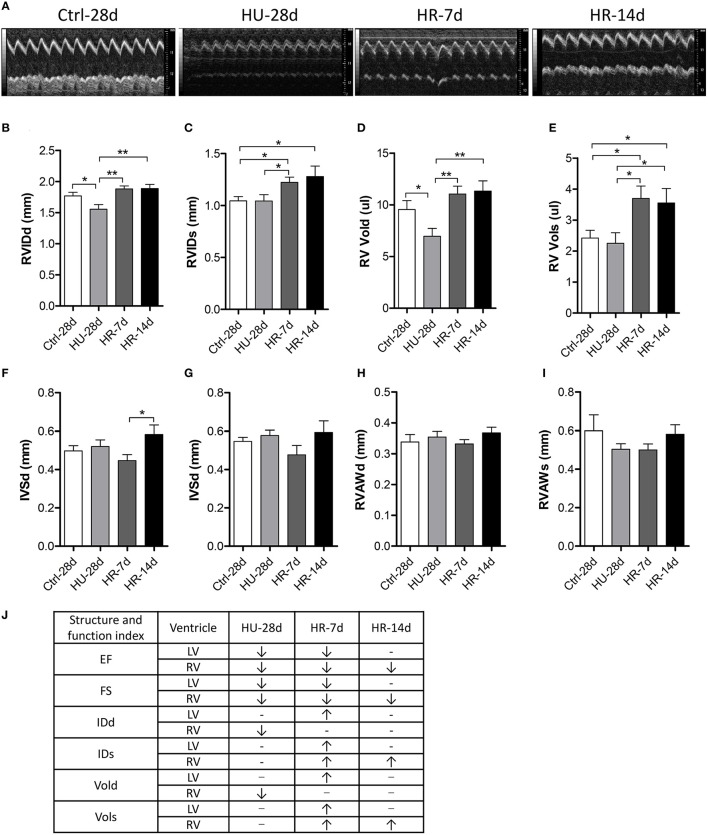
**Transthoracic echocardiography evaluating the right ventricular structure of mice following hindlimb unloading and recovery**. **(A)** Representative M-mode recordings of echocardiography. **(B–I)** Quantitative analysis of the diastolic and systolic right ventricular internal diameter (RVIDd and RVIDs), RV volume (RV Vold and RV Vols), interventricular septal thickness (IVSd and IVSs), and RV anterior wall thickness (RVAWd and RVAWs) of mice by echocardiography following hindlimb unloading and recovery. **(J)** Summary of Structure and function index of LV and RV. Values are means ± SEM. ^*^P < 0.05, ^**^P < 0.01.

To validate the influence of HU and HR in the RV, we performed transthoracic echocardiography to assess the structure and function of the RV following HU and HR (Figure 4A). After 28 days of HU, the end-diastolic RV internal diameter (RVIDd) and the end-diastolic RV volume (RV-Vold) decreased in the HU mice compared with control, but recovered to its normal state in the HR-7d and HR-14d groups (Figures 4B,D). Furthermore, the end-systolic RV internal diameter (RVIDs) and the end-systolic RV volume (RV-Vols) did not change following HU, but increased in the HR-7d and HR-14d groups (Figures 4C,E). The RV anterior wall thickness (RVAWd and RVAWs) and the interventricular septal thickness (IVSd and IVSs) did not change following HU or HR (Figures 4F–I). Together, these data show that HU and HR induce different structural changes in the LV and RV (Figure 4J) and, during the recovery process, cardiac remodeling may be intensified because of reloading.

### HU and HR lead to cardiac remodeling

**Figure 5:**
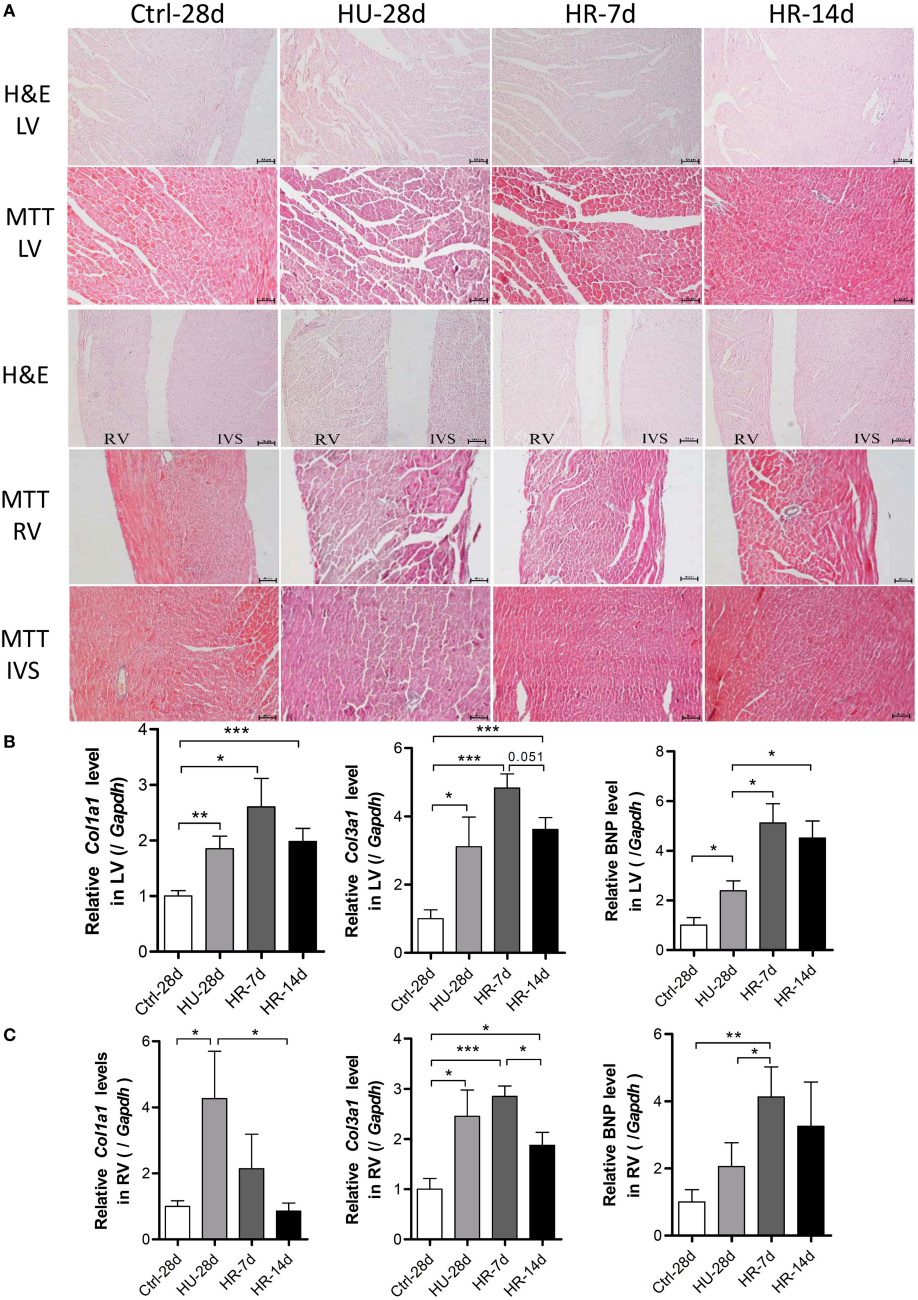
**Hindlimb unloading and reloading lead to cardiac remodeling. (A)** H&E stained sections and MTT stained sections of the left ventricle (LV), right ventricle (RV), and interventricular septum (IVS). **(B,C)** mRNA levels of *Col1a1, Col3a1*, and BNP in the LV and RV were analyzed by qPCR, *n* = 7. H&E, hematoxylin and eosin; MTT, Masson trichrome staining; *Col1a1*, alpha-1 type I collagen; *Col3a1*, alpha-1 type III collagen; BNP, brain natriuretic peptide; qPCR, real-time polymerase chain reaction. Values are means ± SEM (*n* = 7). ^*^P < 0.05, ^**^P < 0.01, ^***^P < 0.001.

To address the influence of HU and HR in the LV and RV, hearts from mice following HU and HR were assessed for changes in morphology and gene expression. Histological analysis showed heart remodeling occurred following HU and HR. In hematoxylin and eosin-stained (HE) sections, gross evidence of edema was easily observed by separation of the myofibers in the LV, the interventricular septum (IVS), and the RV following HU. Recovery to a normal state occurred following 14 days of HR (Figure 5A). Masson trichrome staining (MTT) showed a deeper staining of collagen in the LV, the interventricular septum, and the RV following HU. These changes also recovered after 14 days of reloading. In the HU-28d and HR-7d groups, relative *Col1a1, Col3a1*, and BNP mRNA levels increased in the LV, and recovered, although not significantly (Figure 5B). In the RV, the relative mRNA levels of *Col1a1* and *Col3a1* increased in the HU-28d group, and only the level of *Col1a1* recovered to its normal state in the HR-14d group. The relative mRNA level of BNP increased following HU, and continued to increase after 7 days of HR (Figure 5C). These results demonstrate that HU leads to slight fibrosis and remodeling in both the left and right ventricle, and recovery occurs slowly after 14 days of reloading.

### Changes in HDAC4, AMPK, ERK1/2, and LC3-II activity in the left and right ventricles induced by HU and HR

**Figure 6:**
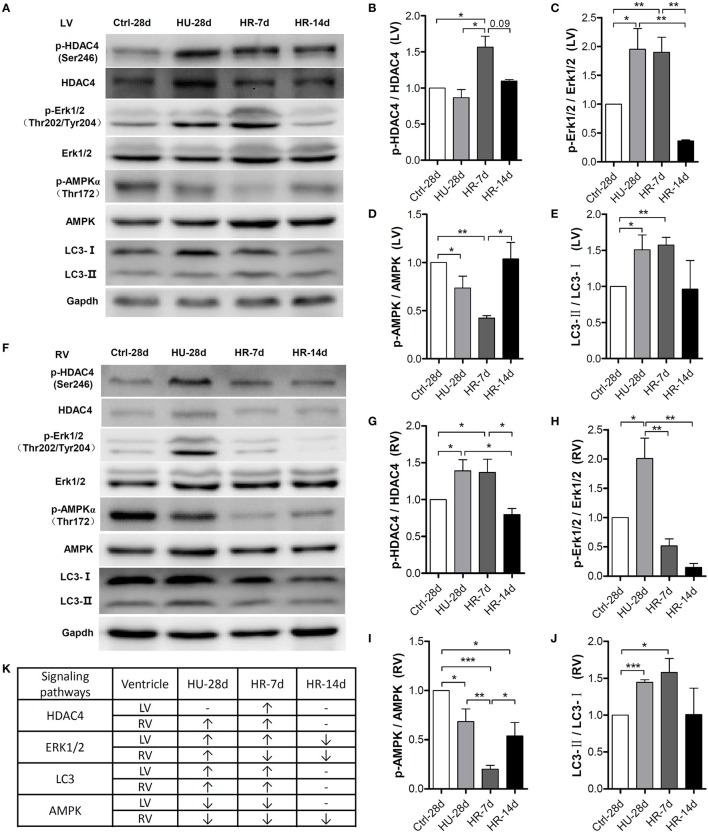
**Activity of signaling pathways in the mouse heart following hindlimb unloading and recovery. (A,F)** Representative western blots of HDAC4 and its phosphorylation at Ser246, AMPKα and its phosphorylation at Thr172, ERK1/2, and its phosphorylation at Thr202/Tyr204, and LC3 of the left ventricle (LV) and right ventricle (RV). Gapdh levels served as a loading control. Quantification of phosphorylation levels normalized to total protein (LC3-II levels normalized to LC3-I levels) of the LV **(B–E)** and RV **(G–J)**. **(K)** Summary of changed signaling molecules. HDAC4, Histone Deacetylase 4; ERK, Extracellular Regulated Protein Kinases; AMPK, AMP-activated Protein Kinase; LC3, Microtubule-associated Protein Light Chain 3. Gapdh, Glyceraldehyde phosphate dehydrogenase. Values are means ± SEM (*n* = 4). ^*^P < 0.05, ^**^P < 0.01, ^***^P < 0.001.

To gain more insight into the signaling pathways involved in the declining function of both the left and right ventricles, we examined several important signaling molecules involved in cardiac remodeling induced by external or intrinsic stimuli. As shown in Figure 6A, quantification of phosphorylation levels normalized to total protein in the LV revealed that HDAC4 phosphorylation at Ser246 did not change following HU but increased following 7 days of reloading, and was fully restored after 14 days. Compared with the control, Erk1/2 phosphorylation at Thr202/Tyr204 increased following HU, remained the higher level during the first 7 days of reloading, and was fully restored after 14 days. The phosphorylation level of AMPK at Thr172 decreased following HU, continued to decrease after 7 days of reloading, and was restored after 14 days. According to the research of Liu et al. (2015), autophagy is involved in HU-induced decline in heart function, so we assessed the change of LC3-II in our model. Quantification of LC3-II levels normalized to LC3-I revealed an increased ratio of LC3-II: LC3-I following HU, and this ratio did not return to its normal state until after 14 days of reloading. The changes of these signaling pathways in RV following HU and HR were also analyzed. As shown in Figure 6B, the level of HDAC4 phosphorylation at Ser246 in the RV increased following HU, however it was not restored to the normal level until 14 days of reloading, which is in contrast to the changes in LV. Phosphorylation of Erk1/2 at Thr202/Tyr204 was up-regulated increased following HU, and recovered after 7 days of reloading. The phosphorylation of AMPK at Thr172 was reduced following HU, and continued to decrease after 7 days of reloading, however, it was not restored to its normal state until 14 days after reloading, which is different from the changes in the LV. The changes of LC3-II: LC3-I was the same as that for the LV.

We also analyzed the changes of mTOR phosphorylation and MuRF1/Atrogin1 levels, which are involved in protein synthesis and degradation pathways, respectively. In LV, As is shown in Figure S1A, mTOR phosphorylation at S2448 was decreased following HU, and was restored following 7 days of reloading. In comparison with the control, the level of Atrogin1, an E3 ubiquitin ligase that mediates proteolysis events during muscle atrophy, obviously increased following HU, and remained much higher level during the first 7 days of reloading, then restored to the normal level after 14 days of reloading. The level of MuRF1, another ubiquitin ligases, was increased in HU group, however, it did not recover to the normal condition even after 14 days of reloading (Figure S1A). In RV, as shown in Figure S1B, the changes of phosphorylation level of mTOR was the same as that for the LV. The levels of Atrogin1 and MuRF1 were both substantially increased following HU, and restored till 14 days of reloading (Figure S1B). The changes of these signaling pathways were closely related to disorders of cardiac function observed both in the LV and RV and the differences between them.

## Discussion

In this study, we report for the first time the differences between left and right ventricular remodeling induced by simulated microgravity and reloading. We also characterize the signaling molecules involved in this cardiac remodeling. Consistent with previous reports, our study indicates that left ventricular function declines following HU but recovers to its normal state after 14 days of reloading. Few studies have focused on the RV, however. We show here that the function of the RV also declines following HU, but does not recover even after 14 days of reloading. In other words, both the left and right ventricle exhibited a decline in function, but recovery of right ventricular function was much more difficult. We demonstrate that pathological remodeling signals, such as HDAC4, were activated following HU and recovered following HR in both the LV and RV. The physiological remodeling signal AMPK was inhibited in both the LV and RV following HU, but only restored in the LV following 14 days of HR.

Several studies have suggested that microgravity or simulated microgravity lead to cardiac remodeling, and result in cardiac deconditioning when reloaded. In humans exposed to 6 weeks of bed rest, LV mass decreased by 8.0 ± 2.2%, RV free wall mass decreased by 10 ± 2.7%, and RV end-diastolic volume decreased by 16 ± 7.9%. After 10 days of spaceflight, LV mass decreased by 12 ± 6.9%. Thus, cardiac atrophy occurs during prolonged horizontal bed rest, but may also occur after short-term spaceflight (Perhonen et al., 2001). Using an experiment with 60 days of sedentary head-down bed rest, one group demonstrated that the reduced LV mass in response to prolonged simulated weightlessness is not simply due to tissue dehydration but rather to true LV remodeling that persists well into recovery (Westby et al., 2016). Previous studies conducted on rats and mice have provided conflicting data. Bigard et al. (1994) demonstrated that LV mass decreased following HU for 21 days in rats. Ray et al. (2001), however, suggested that the mass of the rat heart was unchanged after 28 days of HU. Jennifer et al. (Powers and Bernstein, 2004) also reported that absolute heart weights were not altered significantly after 14 days of tail suspension in mice. Moreover, few studies have focused on right ventricular remodeling induced by space flight or simulated microgravity. In our research, LV mass and structure did not change following 28 days of HU, consistent with some of the previous studies, but RVIDd and RV Vold did decrease, and RV mass trended slightly lower following HU. So, we suggest that the RV is more sensitive than the LV following HU.

Many studies have demonstrated that cardiac remodeling is associated with a decline in heart function induced by microgravity and/or simulated microgravity. However, the changes in heart structure and function during reloading after simulated microgravity are not clear. In our study, heart weight increased significantly following HR for 7 days compared with the HU group, and recovered after 14 days of HR (Figure 1C). The masses of both the LV and RV increased following 7 days of HR, although the changes were not significant (Figures 1E,F). Echocardiography revealed that LVIDd, LVIDs, LV Vold, and LV Vols increased following 7 days of HR, and recovered after 14 days (Figures 3B–E). RVIDs and RV Vols also increased following HR for 7 and 14 days (Figures 4C,E). In summary, both the LV and RV were enlarged following 7 days of HR. These results indicate that simulated microgravity leads to cardiac remodeling, and in the early stages of recovery, reloading may intensify remodeling.

The mammalian heart is a muscle, the fundamental function of which is to pump blood throughout the circulatory system. In response to changed workload, typically caused by pathological or physiological stimulation, the heart undergoes remodeling in an attempt to maintain pump function in the new environment (Maillet et al., 2013). A variety of stimuli can induce the heart to grow or shrink. Exercise, pregnancy, and postnatal growth promote physiologic growth of the heart, while neurohumoral activation, hypertension, and myocardial injury can cause pathologic hypertrophic growth. As with other forms of cardiac remodeling, ventricular atrophy is induced by prolonged weightlessness during space travel, prolonged bed rest, and mechanical unloading with a ventricular assist device (Hill and Olson, 2008). Well-characterized signaling molecules that regulate cardiac remodeling include HDAC4, AMPK, ERK1/2, LC3-II, mTOR, Atrogin1, and MuRF1. HDAC4, a key member of class IIa HDACs (HDACs 4, 5, 7, and 9), is expressed in numerous tissues, and plays an important role in the modulation of biological responses and pathological disorders (Yang and Grégoire, 2005; Backs and Olson, 2006; Wang et al., 2014). Phosphorylated HDAC4 is exported to the cytoplasm from the nucleus, with consequent activation of MEF2 and its downstream target genes involved in pathological cardiac remodeling (Passier et al., 2000; Haberland et al., 2009; Ling et al., 2012). AMPK is a stress-activated kinase which functions as a cellular fuel gauge and master metabolic regulator, and is therefore crucial to cardiac homeostasis (Coughlan et al., 2014). The activation of heart AMPK is associated with the translocation of GLUT4 and phosphorylation of acetyl-CoA carboxylase (ACC), which promote ATP production by stimulating fatty acid oxidation, glucose uptake, and glycolysis (Coven et al., 2003; Maillet et al., 2013). AMPK is important for maintaining the physiological growth of the heart. ERK1/2 belongs to the mitogen-activated protein kinase (MAPK) family, and its activation has been reported to mediate both pathological and physiological cardiac remodeling (Tham et al., 2015). According to the research of Liu et al. (2015), autophagy is involved in HU-induced LV decline in function; LC3-II expression increased in the LV after HU. mTOR is an atypical serine/threonine protein kinase that belongs to the phosphoinositide 3-kinase (PI3K)-related kinase family and interacts with several proteins to form two distinct complexes named mTOR complex 1 (mTORC1) and 2 (mTORC2; Laplante and Sabatini, 2012). In muscle, activation of mTORC1 can stimulate protein synthesis to drive muscle hypertrophy (Philp et al., 2011). MuRF-1 and Atrogin 1 are two identified muscle specific ubiquitin ligases, which have been shown to be upregulated prior to the onset of atrophy in multiple models of muscle wasting (Bodine et al., 2001). In this study, we detected these molecular signals in the LV and RV after HU and HR, and we explored the molecular mechanism of LV and RV remodeling induced by simulated microgravity and recovery. We found that the phosphorylation of HDAC4 at Ser246 was upregulated following HU for 28 days. This phosphorylation remained high in the RV (Figures 6F,G), and increased in the LV following 7 days of HR (Figures 6A,B). Meanwhile, the phosphorylation levels of ERK1/2 increased in both the LV and RV following HU for 28 days (Figures 6A,C,F,H), and further increased in the LV following 7 days of HR. Our results also showed that the ratio of LC3-II:LC3-I increased in both the LV and RV following 28 days of HU and 7 days of HR (Figures 6A,E,F,J). Autophagy was activated in both the LV and RV, consistent with previous reports (Liu et al., 2015). These results indicate that both HU-simulated microgravity and reloading can activate pathological cardiac remodeling signaling pathways, which can initiate the expression of fetal genes in both the LV and RV, and ultimately lead to cardiac remodeling. Following HU, The phosphorylation of mTOR at S2448 was decreased both in LV and RV (Figure S1), protein synthesis pathway was inhibited. The levels of Atrogin1 and MuRF1 were increased in both LV and RV following 28 days of HU and 7 days of HR (Figure S1), which suggest that ubiquitin-proteasome system was activated both in LV and RV. The changes of these proteins contributed to the cardiac remodeling. The phosphorylation level of AMPK at Thr172 decreased following 28 days of HU and continued to decrease following 7 days of HR in both the LV and RV (Figures 6A,D,F,I). Moreover, the phosphorylation of AMPK returned to a normal level in the LV following 14 days of HR. This did not occur in the RV, however. Interestingly, levels of AMPK phosphorylation were consistent with the functional changes in both the LV and RV. The physiological remodeling signal AMPK decreased following HU in both the LV and RV, and did not return to its normal state in the RV following 14 days of HR. This may at least partially explain the different responses of the RV and LV following HU and HR.

This study provides evidence of the differences in the responses of the LV and RV under simulated microgravity and the signaling molecules involved in this process. We found that simulated microgravity leads to cardiac remodeling, and this remodeling could be reversed. In the early stages of recovery, reloading may intensify cardiac remodeling. Moreover, it is more difficult to restore the changes in the RV compared with the LV. Finally, we identified that following HU and HR, pathological remodeling signals, such as HDAC4, were activated, and physiological remodeling signals, such as AMPK, were inactivated in both the LV and RV, which led to cardiac remodeling and decline of heart function (Figure 6K).

## Author contributions

YXL and SL conceived the study, GZ performed the experiment with support from WS, DC, QX, HC, and HL; GZ, YHL, JL, and DZ analyzed and interpreted the results; JS, XW, GK, and QL provided intellectual contribution; GZ, SL wrote the manuscript with the assistance of XJ, HS, and XY; YXL, SL, and YHL revised the manuscript and gave final approval of the submitted manuscript. All authors have reviewed and approved the final manuscript. The English in this document has been checked by at least two professional editors, both native speakers of English. For a certificate, please see: http://www.textcheck.com/certificate/UQCwt5.

## Funding

This work was supported by the National Natural Science Foundation of China (No. 31300698, 31271225, and 31325012) and the Grant of State Key Lab of Space Medicine Fundamentals and Application (No. SYFD130051833, SYFD140041803).

### Conflict of interest statement

The authors declare that the research was conducted in the absence of any commercial or financial relationships that could be construed as a potential conflict of interest. The reviewer [JA] and handling Editor declared their shared affiliation, and the handling Editor states that the process nevertheless met the standards of a fair and objective review

## Supplementary material

Figure S1 **The changes of protein levels involved in protein synthesis and proteolysis**. Representative western blots of mTOR and its phosphorylation at Ser2448, Atrogin1, and MuRF1 of the left ventricle **(A)** and right ventricle **(B)**. Gapdh levels served as a loading control. mTOR, Mammalian Target of Rapamycin; MuRF-1, Muscle Ring Finger 1.
