# Supplementary figures and images for: Simulated Microgravity and Recovery-Induced Remodeling of the Left and Right Ventricle
Source: Front Physiol. 2016 Jun 29;7:274. doi: 10.3389/fphys.2016.00274 (PMC4925715; doi:10.3389/fphys.2016.00274)

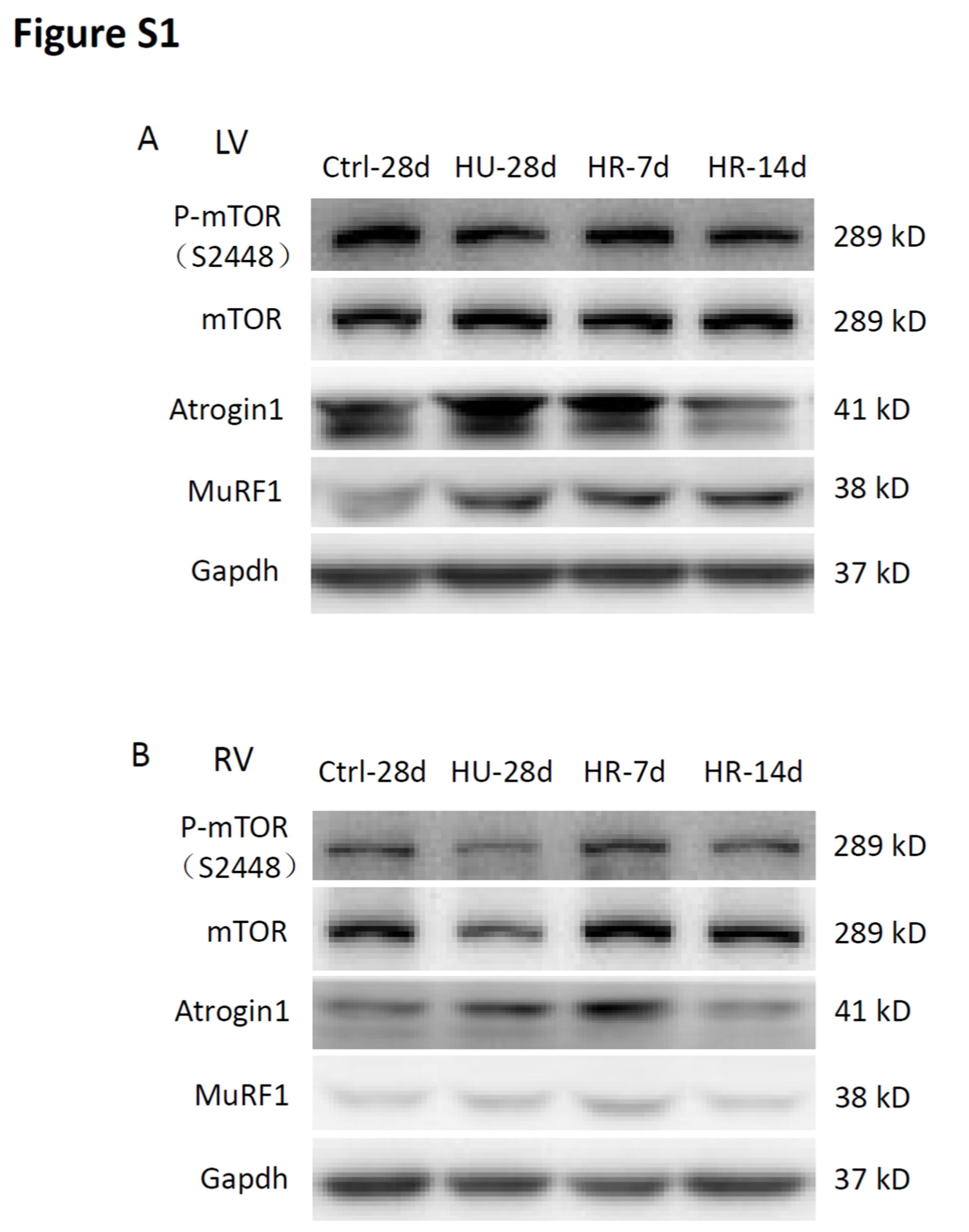

Supplement: Figure S1 — The changes of protein levels involved in protein synthesis and proteolysis. Representative western blots of mTOR and its phosphorylation at Ser2448, Atrogin1, and MuRF1 of the left ventricle (A) and right ventricle (B). Gapdh levels served as a loading control. mTOR, Mammalian Target of Rapamycin; MuRF-1, Muscle Ring Finger 1. [file Image1.tif]
